# Echocardiographic predictors of intraoperative right ventricular dysfunction: a 2D and speckle tracking echocardiography study

**DOI:** 10.1186/s12947-019-0161-3

**Published:** 2019-06-07

**Authors:** Lisa Q. Rong, Brian Yum, Christiane Abouzeid, Maria Chiara Palumbo, Lillian R. Brouwer, Richard B. Devereux, Leonard N. Girardi, Jonathan W. Weinsaft, Mario Gaudino, Jiwon Kim

**Affiliations:** 1 Department of Anesthesiology, Weill Cornell Medicine/New York Presbyterian, New York, NY USA; 2 Department of Medicine/Cardiology Division, Weill Cornell Medical College, 525 East 68th Street, New York, NY 10021 USA; 3 Department of Cardiothoracic Surgery, Weill Cornell Medicine/New York Presbyterian, New York, NY USA

**Keywords:** Right ventricular function, Cardiac surgery, 2D speckle tracking, Intraoperative transesophageal echocardiography

## Abstract

**Background:**

Intraoperative or post procedure right ventricular (RV) dysfunction confers a poor prognosis in the post-operative period. Conventional predictors for RV function are limited due the effect of cardiac surgery on traditional RV indices; novel echocardiographic techniques hold the promise to improve RV functional stratification.

**Methods:**

Comprehensive echocardiographic data were collected prospectively during elective cardiac surgery. Tricuspid annular plane systolic excursion (TAPSE), peak RV systolic velocity (S′), and RV fractional area change (FAC) were quantified on transesophageal echo (TEE). RV global and regional (septal and free wall) longitudinal strain was quantified using speckle-tracking echo in RV-focused views. Two intraoperative time points were used for comparison: pre-sternotomy (baseline) and after chest closure.

**Results:**

The population was comprised of 53 patients undergoing cardiac surgery [15.1% coronary artery bypass graft (CABG) only, 28.3% valve only, 50.9% combination (e.g. valve/CABG, valve/aortic graft) surgeries], among whom 38% had impaired RV function at baseline defined as RV FAC < 35%. All conventional RV functional indices including TAPSE, S′ and FAC declined immediately following CPB (1.5 ± 0.3 vs.1.1 ± 0.3 cm, 8.0 ± 2.1 vs. 6.2 ± 2.5 cm/s, 36.8 ± 9.3 vs. 29.3 ± 10.6%; *p* < 0.001 for all). However, left ventricular (LV) and RV hemodynamic parameters remained unchanged (LV ejection fraction (EF): 56.8 ± 13.0 vs. 55.8 ± 12.9%; *p* = 0.40, pulmonary artery systolic pressure (PASP): 26.5 ± 7.4 vs 27.3 ± 6.7 mmHg; *p* = 0.13). Speckle tracking echocardiographic data demonstrated a significant decline in RV global longitudinal strain (GLS) [19.0 ± 6.5 vs. 13.5 ± 6.9%, *p* < 0.001]. Pre-procedure FAC, GLS and free wall strain predicted RV dysfunction at chest closure (34.7 ± 9.1 vs. 41.6 ± 8.1%, *p* = 0.01, 17.7 ± 6.5 vs. 21.8 ± 5.4%; *p* = 0.03, 20.3 ± 6.4 vs. 24.2 ± 5.8%; *p* = 0.04), whereas traditional linear RV indices such as TAPSE and RV S′ at baseline had no impact on intraoperative RV dysfunction (*p* = NS for both).

**Conclusions:**

Global and regional RV function, as measured by 2D indices and strain, acutely decline intraoperatively. Impaired RV strain is associated with intraoperative RV functional decline and provides incremental value to traditional RV indices in predicting those who will develop RV dysfunction.

## Background

RV dysfunction is common; it occurs in nearly one fourth of patients who undergo cardiac surgery. Intraoperative or post procedure RV dysfunction is linked to high post-operative mortality [1, 2]. However, RV assessment can be challenging due to its anterior location and complex crescent-shaped geometry, which pose particular imaging difficulties in the intraoperative setting. Prior studies have shown that commonly used indices of RV function such as tricuspid annular plane systolic excursion (TAPSE) can be altered by pericardiotomy itself [3, 4]. For example, decrements in longitudinal shortening have been shown to result in gains in transverse shortening [5] thereby further limiting traditionally used longitudinal measures of RV performance, TAPSE and RV S′. In addition to these limitations, longitudinal RV contraction in the intraoperative setting has been shown to reflect geometric changes related to cardiac surgery itself, rather than due to true functional decline [6].

Myocardial deformation via two-dimensional speckle tracking echocardiography (2D STE) provides incremental value in prognostic stratification as compared to traditional indices in a wide range of cardiac conditions including heart failure, coronary artery disease and pulmonary hypertension [7–9]. However, its role in the intraoperative setting has yet to be established. In particular, the angle-independent nature of speckle tracking has the potential to overcome limitations of 2D linear indices of TAPSE and S′ and is thereby uniquely suited for RV assessment in this setting. In addition, RV deformation has been shown to detect subclinical RV dysfunction and therefore may be of utility in predicting post-operative outcomes [10, 11]. The goals of this study were: 1. to address the acute intraoperative impact on RV function by using both standard 2D and deformation echo indices; and 2. to test the performance of conventional and deformation indices to predict intraoperative RV dysfunction.

## Methods

### Study population

Patients were enrolled prospectively from 11/2017 to the present as part of an established protocol (PALACS Trial) at Weill Cornell Medical College [12].

### Imaging protocol

#### Echocardiography

Comprehensive TEEs were performed prior to sternotomy (baseline) and after chest closure (post procedure) using commercial equipment (GE Vivid 7 [GE Healthcare, Madison, WI]) and Phillips iE33 [Phillips Medical Systems, Andover, MA] echo systems). TEEs were interpreted by experienced investigators within a high-volume laboratory, for which expertise and reproducibility for quantitative RV indices have been validated [13, 14].

#### 2D TEE

RV systolic function was quantified using tricuspid annular plane systolic excursion (TAPSE), right ventricular systolic myocardial velocity (RV-S′) and fractional area of change (FAC), which were acquired in accordance with consensus guidelines [15]. TAPSE was measured on M-mode as the systolic excursion of the lateral tricuspid annulus along its longitudinal plane and RV-S′ on tissue Doppler as the peak tricuspid annular velocity of excursion. FAC was measured via planimetry of end-diastolic and end-systolic contours in mid-esophageal 4-chamber orientation. The established FAC cut off (< 35%) was used to identify post procedure (intraoperative) RV dysfunction [16]. Right atrial area was calculated by planimetry in end systole in mid-esophageal 4-chamber view and volume was quantified by area-length method.

### Speckle-tracking strain analysis

2D echocardiographic images were analyzed offline for deformation analysis (2D CPA; TomTec Imaging Systems). Strain measurements were performed by an investigator experienced in the interpretation of echocardiographic images, blinded to the results of the 2D measurements. Each image was acquired in the mid-esophageal four-chamber view of the whole RV cavity and with a frame rate > 50 Hz for analysis of RV strain. The RV end-diastolic endocardial border was manually traced and tracked automatically frame-by-frame throughout the cardiac cycle. Endocardial contour was manually adjusted when necessary to optimize tracking.

### Statistical analysis

Comparisons between groups were made using Student’s t-test (expressed as mean ± standard deviation) for continuous variables. Categorical variables were compared using Chi-square or, when fewer than 5 expected outcomes per cell, Fisher’s exact test. Bivariate correlation coefficients, as well as regression analyses were used to evaluate univariable associations between continuous variables. Multivariate modeling was performed via logistic regression and echo indices were tested as continuous variables. Inter-rater and intra reliability was calculated using the intraclass-correlation coefficient. Statistical calculations were performed using SPSS 22.0 (SPSS Inc. [Chicago, IL]). Two-sided *p* < 0.05 was considered indicative of statistical significance.

## Results

The study population included 53 patients who underwent cardiac surgery with dedicated TEE for RV assessment at baseline and post procedure. Intraoperative RV dysfunction was defined as RV FAC < 35% post procedure. In this population, 38% had RV dysfunction at baseline and over two thirds (70%) had some degree of RV dysfunction post procedure. Table 1 details clinical characteristics of the population, stratified by the presence or absence of TEE-verified intraoperative RV dysfunction. As shown, 15.1% coronary artery bypass graft (CABG) only, 28.3% valve only, 50.9% combination (e.g. valve/CABG, valve/aortic graft) surgeries; operation type had no impact on RV function. With respect to clinical characteristics, there were no differences between cardiovascular risk factors. Similarly, there were no differences in symptom status pre-operatively as characterized via New York Heart Association (NYHA) class (*p* = 0.15).

**Table 1:** Clinical Characteristics

	Overall (*n* = 53)	RV Dysfunction +^a^ (*n* = 37)	RV Dysfunction – (*n* = 16)	*p*
Age (years)	63 ± 11	61 ± 11	67 ± 10	0.09
Male gender	69.8% (37)	75.7% (28)	54.3% (9)	0.20
BMI	27.8 ± 4.6	28.2 ± 4.7	27.2 ± 4.3	0.60
CV Risk Factors				
Hypertension	78.8% (41)	73.0% (27)	87.5% (14)	0.47
Diabetes	11.3% (6)	13.5% (5)	6.3% (1)	0.66
Prior Myocardial Infarction	9.4% (5)	13.5% (5)	0.0% (0)	0.31
Operation				
CABG Only	15.1% (8)	18.9% (7)	6.3% (1)	0.41
Valve Surgery Only	28.3% (15)	29.7% (11)	25.0% (4)	1.00
Concurrent Aortic Surgery	39.6% (21)	37.8% (14)	43.8% (7)	0.69
Aortic Surgery Only	3.8% (2)	5.4% (2)	0% (0)	1.00
Combination	50.9% (27)	45.9% (17)	62.5% (10)	0.27
Other	1.9% (1)	0% (0)	6.3% (1)	0.30
NYHA Class (I/II/III/IV)	72% (36)/ 18% (9)/ 10% (5)/ 0% (0)	66% (23)/ 23% (8)/ 11% (4)/ 0% (0)	86% (13)/ 7% (1)/ 7% (1)/ 0% (0)	0.15

*BMI* body mass index, *CV* cardiovascular, *CABG* coronary artery bypass graft, *NYHA* New York Heart Association

^a^Categorized based on established echo cut off FAC < 35% (post chest closure)

**Fig. 1:**
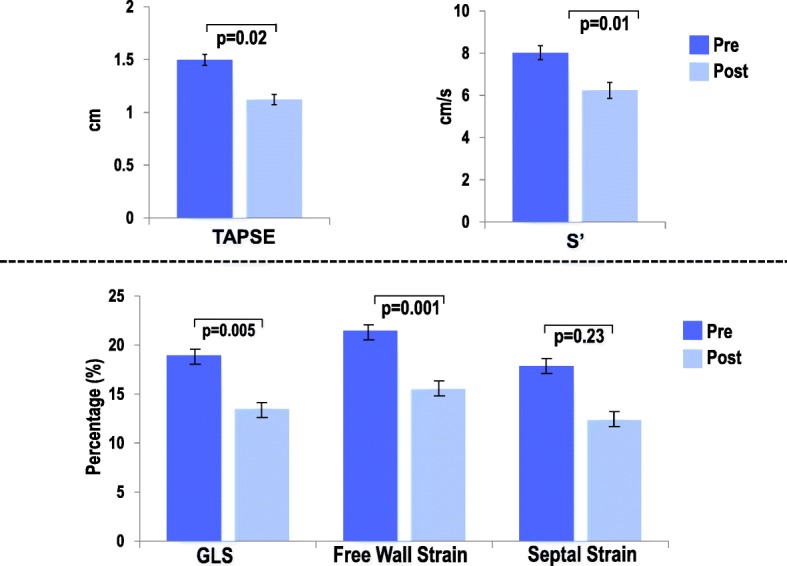
RV parameters including linear and deformational indices decline intraoperatively. GLS decline is driven predominantly by impairments in free wall strain as there is no difference in interventricular septal deformation post procedure (*p* = 0.23)

**Fig. 2:**
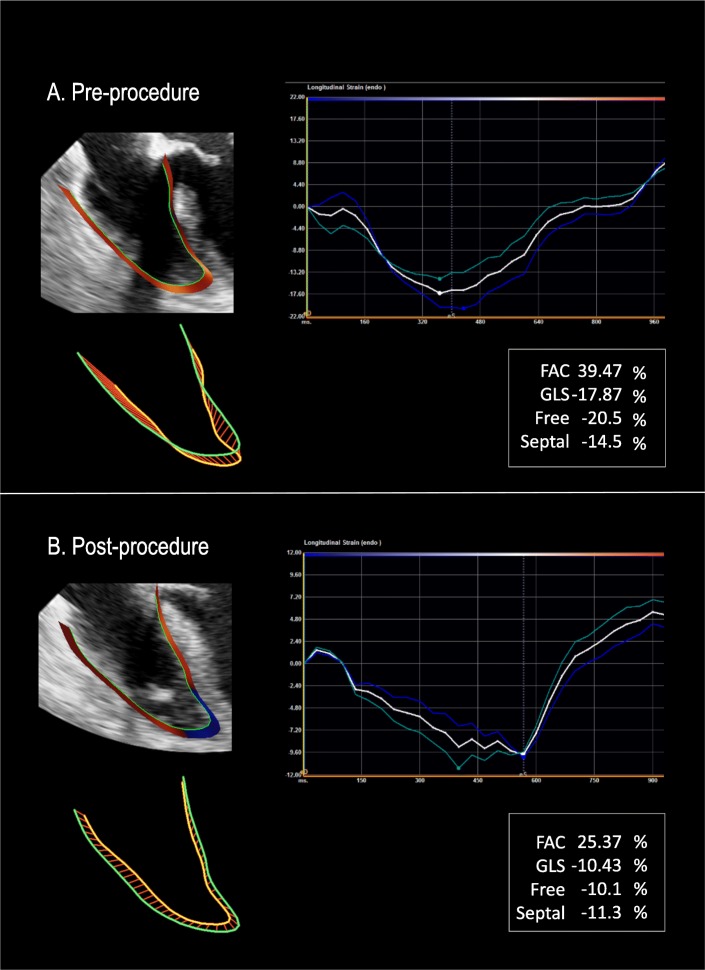
**a** Representative example of pre-procedural TEE-derived RV strain analysis acquired in a patient undergoing cardiac surgery. **b** Post-procedural TEE analysis as acquired in the same patient: As shown, despite normal conventional index (FAC), this patient had impaired RV strain which is a risk factor for post procedure RV dysfunction

Table 2 demonstrates LV, RA, RV and hemodynamic indices at baseline and post procedure in relation to RV dysfunction. As shown, RV echocardiographic indices including conventional parameters and global longitudinal strain declined after chest closure (*p* < 0.05 for all). In support of the concept that RV functional decline is driven predominantly by effects on the RV free wall, there was no significant change in post procedure interventricular septal deformation (*p* = 0.23) (Fig. 1). When examining predictors for RV dysfunction post chest closure, baseline FAC, GLS and free wall strain were impaired in those patients with intraoperative RV dysfunction (34.7 ± 9.1 vs. 41.6 ± 8.1%; *p* = 0.01, 17.7 ± 6.5 vs. 21.8 ± 5.4% *p* = 0.03, 20.3 ± 6.4 vs. 24.2 ± 5.8%; *p* = 0.04, respectively). On the other hand, traditional linear RV indices such as TAPSE and S′ had no impact on post chest closure RV dysfunction (1.4 ± 0.3 vs. 1.6 ± 0.4 and 7.9 ± 2.4 vs. 8.5 ± 2.4; *p* = NS for both). Figure 2 represents a patient who developed intraoperative RV dysfunction with baseline impaired RV deformation and normal function as defined as FAC > 35%.

**Table 2:** Echocardiographic and Hemodynamic Parameters

	Overall (*n* = 53)	RV Dysfunction +^a^ (*n* = 37)	RV Dysfunction – (*n* = 16)	*p*
Baseline				
LV				
EDV (mL/m^2^)	104.1 ± 45.5	108.4 ± 39.6	93.1 ± 58.3	0.31
ESV (mL/m^2^)	48.0 ± 31.3	51.6 ± 30.9	39.0 ± 31.7	0.22
EF (%)	56.8 ± 13.0	55.9 ± 14.0	59.2 ± 10.0	0.45
RV				
EDA (cm^2^)	19.3 ± 6.9	19.4 ± 6.7	19 ± 7.5	0.85
ESA (cm^2^)	12.2 ± 4.9	12.6 ± 4.6	11.2 ± 5.4	0.34
TAPSE (cm)	1.5 ± 0.4	1.4 ± 0.3	1.6 ± 0.4	0.14
S′ (cm/s)	8.0 ± 2.1	7.9 ± 2.4	8.5 ± 2.4	0.48
GLS (%)	−19.0 ± 6.5	−17.7 ± 6.5	−21.8 ± 5.4	**0.03**
Septal Strain (%)	−17.9 ± 5.9	−16.9 ± 5.5	−20.0 ± 6.4	0.08
Free Wall Strain (%)	−21.5 ± 6.4	−20.3 ± 6.4	−24.2 ± 5.8	**0.04**
FAC (%)	36.8 ± 9.3	34.7 ± 9.1	41.6 ± 8.1	**0.01**
RA				
Area (cm^2^)	14.9 ± 4.9	14.4 ± 4.8	16 ± 5.3	0.35
Length (cm)	3.9 ± 0.8	3.8 ± 0.7	4.1 ± 0.9	0.33
Volume Index (mL/m^2^)	30.3 ± 13.9	28.6 ± 13.1	34.4 ± 15.5	0.21
Hemodynamics				
LV EDP (mmHg)	13.8 ± 4.6	13.3 ± 4.0	15.3 ± 5.9	0.23
PASP (mmHg)	26.5 ± 7.4	27.0 ± 7.3	25.5 ± 7.9	0.50
PADP (mmHg)	10.5 ± 4.0	10.9 ± 3.9	9.8 ± 4.1	0.34
RAP (mmHg)	7.2 ± 2.7	7.6 ± 2.8	6.4 ± 2.6	0.13
RV CO (L)	4.6 ± 1.8	4.5 ± 1.9	4.8 ± 1.6	0.67
Post procedure				
LV				
EDV (mL/m^2^)	79.7 ± 30.3	82.2 ± 27.2	73.1 ± 37.5	0.36
ESV (mL/m^2^)	37.0 ± 20.5	38.7 ± 19.2	32.8 ± 23.6	0.39
EF (%)	55.8 ± 12.9	54.1 ± 13.0	60.0 ± 12.2	0.17
RV				
TAPSE (cm)	1.1 ± 0.3	1.1 ± 0.3	1.1 ± 0.4	0.94
S′ (cm/s)	6.2 ± 2.5	6.0 ± 2.3	6.7 ± 2.9	0.41
GLS (%)	−13.5 ± 6.9	−10.1 ± 4.3	−21.2 ± 5.3	**< 0.001**
Septal Strain (%)	−12.4 ± 6.6	−9.9 ± 5.2	−18.2 ± 5.9	**< 0.001**
Free Wall Strain (%)	−15.5 ± 7.8	−12.4 ± 5.6	−22.8 ± 7.6	**< 0.001**
FAC (%)	29.3 ± 10.6	24.0 ± 6.9	41.7 ± 6.6	**< 0.001**
Hemodynamics				
PASP (mmHg)	27.3 ± 6.7	27.8 ± 6.6	26.1 ± 6.7	0.39
PADP (mmHg)	11.2 ± 4.3	11.5 ± 4.3	10.6 ± 4.3	0.49
RAP (mmHg)	8.5 ± 3.0	8.8 ± 3.0	7.9 ± 3.1	0.37
RV CO (L)	5.5 ± 1.3	5.5 ± 1.3	5.5 ± 1.3	0.98

*LV* left ventricle, *RV* right ventricle, *RA* right atrium, *EDV* end diastolic volume, *EDS* end systolic volume, *EF* ejection fraction, *EDA* end diastolic area, *ESA* end systolic area, *TAPSE* tricuspid annular plane systolic excursion, *S′* peak systolic velocity, *GLS* global longitudinal strain, *FAC* fractional area of change, *EDP* end diastolic pressure, *PASP* pulmonary artery systolic pressure, *PADP* pulmonary artery diastolic pressure, *RAP* right atrial pressure, *CO* cardiac output. Bold values indicate *p* < 0.05

^a^Categorized based on established echo cut off FAC < 35% (post chest closure)

Regarding LV parameters, there were no differences in baseline or post chest closure LV volumes and LV ejection fraction when stratified by intraoperative RV dysfunction. This finding adds to the concept that intraoperative RV dysfunction occurs independently of LV performance. Hemodynamic indices, including pre and post pulmonary artery (PA) and right atrial (RA) pressures, also had no association with development of RV dysfunction after chest closure. Table 3 reports multivariate analysis concerning RV strain components for prediction of RV dysfunction. As shown, baseline free wall RV strain was independently associated with post-procedure RV dysfunction (OR 1.24 [CI 1.01 to 1.53] *p* = 0.04) even after controlling for septal wall strain. These results indicate that post procedure RV dysfunction is closely linked to altered RV free wall contraction rather than interventricular septal function. Table 4 shows the intra-observer and interobserver measurements for 15 cases as well as the intraclass coefficient. As shown, the intraclass correlation coefficient ranged from 0.92 between measurements and 0.85 between observers.

**Table 3:** Multivariate Regression for Post Procedure (Intraoperative) RV Dysfunction

Variable	Univariate Regression		Multivariate Regression Chi Square = 25.3*, p* < 0.001	
	Odds Ratio (95% Confidence Interval)	*p*	Odds Ratio (95% Confidence Interval)	*p*
Free Wall Strain	1.33 (1.13–1.56)	**< 0.001**	1.24 (1.01–1.53)	**0.04**
Septal Strain	1.29 (1.12–1.49)	**< 0.001**	1.10 (0.90–1.34)	0.36

Bold values indicate *p* < 0.05

**Table 4:** Reproducibility

	Inter-observer Reproducibility			Intra-observer Reproducibility		
	Mean ± SD	Intraclass Correlation Coefficient	Limits of Agreement	Mean ± SD	Intraclass Correlation Coefficient	Limits of Agreement
RV GLS	−0.9 ± 4.5	0.85	−9.6 to 7.9	0.1 ± 3.1	0.92	−6.0 to 6.3

## Discussion

The primary aim of this study was to characterize and predict the acute intraoperative change in RV function using established deformational RV techniques. The findings of this study are as follows: 1. RV dysfunction occurs in over two thirds of patients undergoing cardiac surgery and is associated with impaired RV global longitudinal strain but not conventional 2D RV parameters at baseline. 2. RV functional decline is predominantly associated with impairments in RV free wall deformation rather than that of the interventricular septum. 3.Whereas deformational indices predict intraoperative RV dysfunction, traditional linear RV indices had no association with post-operative RV dysfunction.

While RV function is an important determinant of cardiac surgical outcomes, there are few known predictors of acute intraoperative dysfunction. Studies demonstrate that RV FAC < 35% is associated with the greatest risk of post-operative mortality [2, 17] and that among patients with preserved RV FAC, those with abnormal longitudinal strain are at higher risk for post-operative mortality [18]. Despite our finding that all linear indices decreased at the time of chest closure, only baseline deformation parameters, which included GLS and free wall strain, predicted intraoperative RV dysfunction. This suggests that RV strain may be an important index to stratify RV dysfunction risk intraoperatively, and may have important clinical implications. Regarding prevalence of RV dysfunction pre-operatively, we found that a large number of patients had RV dysfunction at time of surgery (38%). This prevalence is similar to that reported in the literature: for example, Lella et al. showed that CMR-evidenced RV dysfunction occurred in 44% (48/109) among patients undergoing elective CABG and valve surgery [19]. Similarly, Haddad et al. demonstrated echo-evidenced RV dysfunction to occur in 22% (11/50) patients undergoing aortic or mitral valve surgery [17]. Our population, the majority of whom are undergoing CABG and/or valve surgery, had similar high prevalence of RV dysfunction pre-operatively.

Previous studies demonstrate a decline in RV function measured by 2D indices including TAPSE and S′ with transthoracic echocardiography (TTE) 3–6 months after surgery. This may be the result of intraoperative factors such as ischemia, pericardial opening, and myocardial stunning secondary to poor protection during CPB [20]. However, other studies report that RV systolic function was preserved after cardiac surgery, as measured by three-dimensional (3D) TEE and 3D RVEF. Interestingly, Unsworth et al. evaluated TAPSE and S′ after pericardial opening and found that these declined acutely intraoperatively, which supports the hypothesis that a geometric, rather than a functional, change occurs to the RV during surgery [3]. Similar to the above finding, preservation of FAC intraoperatively has been linked to a decrease in TAPSE in the setting of increased right-sided filling pressures after CPB [21]. To our knowledge, this is the most comprehensive study of changes in RV systolic function intraoperatively with correlation of changes in 2D indices with regional and global RV strain. We demonstrate that intraoperative RV systolic function significantly declines across all parameters; in particular, GLS significantly predicts subnormal RV function at the end of the surgery. Our TEE measurements were taken before sternotomy and after chest closure, which suggests that changes in RV indices were due to functional, rather than geometric differences.

At present, 2D STE is a relatively new imaging technique that is not routinely used intraoperatively. Unlike Doppler interrogation, strain measured by 2D is angle independent. Therefore, it may be more accurate and easily applied than tissue Doppler interrogation (TDI). However, 2D STE is dependent on image quality and has lower temporal resolution than TDI. While 2D STE is used to demonstrate subclinical LV dysfunction independent of changes in ejection fraction, it is not yet routinely used to characterize RV dysfunction. Our study demonstrates that it is feasible to use strain as a comparable index to predict RV function in the intraoperative period. We found that free-wall strain, septal strain, global strain, and baseline deformation predicted intraoperative dysfunction [20].

Our study had several limitations. First, 3D RV imaging was not performed routinely as part of this protocol and therefore the effect of cardiac surgery on RV volumes and global EF is not assessed here. Second, the impact of RV dysfunction on post-operative morbidity and mortality is not assessed though it should be noted that intraoperative RV dysfunction has been linked to adverse outcomes in prior studies [1, 2]. Lastly, this study tested immediate intraoperative RV function rather than RV function over time. Future studies evaluating long-term effects of cardiac surgery on the RV are warranted.

## Conclusions

Global and regional RV function, as measured by 2D indices and strain, acutely decline intraoperatively. Moreover, 2D STE of the RV at baseline predicts intraoperative RV dysfunction whereas conventional techniques do not. These findings add to the growing body of literature demonstrating that RV function is significantly impaired during cardiac surgery and suggests that deformational imaging provides incremental value in predicting those who will develop RV dysfunction. The ability to predict this dysfunction could help identify those patients in whom support is needed intraoperatively.

## Data Availability

The datasets generated and/or analyzed during the current study are not publicly available due to institutional restrictions from the Weill Cornell medicine institutional review board.

## Abbreviations

2D STE: Two-dimensional speckle tracking echocardiography
3D: Three-dimensional
BMI: Body mass index
CABG: Coronary artery bypass graft
CPB: Cardiopulmonary bypass
CV: Cardiovascular
EDS: End systolic volume
EDV: End diastolic volume
EF: Ejection fraction
FAC: Fractional area change
GLS: Global longitudinal strain
LV: Left ventricular
NYHA: New York Heart Association
PADP: Pulmonary artery diastolic pressure
PALACS: Posterior Left pericardiotomy for the prevention of postoperative Atrial fibrillation after Cardiac Surgery
PASP: Pulmonary artery systolic pressure
RAP: Right atrial pressure
RV: Right ventricular
S′: Peak systolic velocity
TAPSE: Tricuspid annular plane systolic excursion
TDI: Tissue Doppler interrogation
TEE: Transesophageal echo
TTE: Transthoracic echocardiography
